# A randomized pilot study of the efficacy and safety of loading ticagrelor in acute ischemic stroke

**DOI:** 10.1007/s10072-022-06525-7

**Published:** 2022-11-30

**Authors:** Hany M. Aref, Hala El-Khawas, Ahmed Elbassiouny, Hossam M. Shokri, Mohamed G. Zeinhom, Tamer M. Roushdy

**Affiliations:** 1 Neurology Department, Faculty of Medicine, Ain Shams University, Al Khalifa Elmamon St., Cairo, Egypt; 2 Neurology Department, Faculty of Medicine, Kafr El-Sheikh University, Elgeish St., Kafr El-Sheikh, Egypt

**Keywords:** Ticagrelor, Egypt, Acute ischemic stroke, Ticagrelor versus aspirin

## Abstract

**Background:**

Ticagrelor is one of the most recent antiplatelet drugs to be approved to treat ischemic heart disease. Its efficacy may exceed aspirin in improving clinical outcomes in patients with acute ischemic stroke who are ineligible for rt-PA.

**Objectives:**

We evaluated the safety regarding hemorrhagic complications (as a primary endpoint) and the efficacy (as a secondary endpoint) of a 180-mg loading dose of ticagrelor given within 9 h from the onset of the first-ever non-cardioembolic ischemic stroke.

**Methods:**

We conducted our study on patients aged 18–75 years who presented with their first clinically manifested non-cardioembolic ischemic stroke and were recruited from the emergency department OF Kafr El-Sheik University Hospitals, Egypt.

Eligible patients randomly received ticagrelor or aspirin loading and maintenance doses. Screening, randomization, and initiation of treatment all occurred within the first 9 h of stroke onset.

**Results:**

Eighty-five patients received ticagrelor, and 84 received aspirin. Patients who received ticagrelor had a better clinical outcome in terms of NIHSS improvement at 2 days and 1 week of discharge and a favorable mRS score after 1 week of discharge and at 90-day follow-up. There was no significant difference between the two groups regarding hemorrhagic adverse effects.

**Conclusion:**

This pilot study found that ticagrelor had a better clinical outcome than aspirin based on NIHSS and mRS in acute ischemic stroke patients who received it within 9 h from symptom onset and had a shorter hospital stay duration. Ticagrelor was non-inferior to aspirin regarding hemorrhagic complications.

**Trial registration:**

We registered our trial on ClinicalTrials.gov, named after “ticagrelor versus aspirin in ischemic stroke,” and with a clinical trial number (NCT03884530)—March 21, 2019.

## Background

Stroke is one of the leading causes of long-term disability. It is the second most typical cause of mortality globally. The burden of stroke is much higher in developing countries, accounting for 66% of worldwide strokes [1].

rt-PA is still the only approved treatment for acute stroke. Nevertheless, its accessibility is still low in not only developing countries but also high-income countries due to derangements in supply chains related to war and lack of materials. For this reason, there is an ongoing need for using a loading dose of new antiplatelet agents, which may provide better results for acute stroke patients who are ineligible for rt-PA  [2].

Ticagrelor is a new antiplatelet medication that acts by reversible blockade of the P2Y12 subtype of adenosine diphosphate (ADP) receptor. It was approved by the US Food and Drug Administration (FDA) as a treatment for acute coronary syndromes in 2011, and in 2015, it received approval as a maintenance treatment in patients with a history of a heart attack [3, 4], and in 2020 it was approved by FDA in ischemic stroke secondary prevention based on the results of the THALES trial [5].

Our trial differs from other ticagrelor studies as it focused on comparing the safety and efficacy of ticagrelor versus aspirin within the first 9 h of a first-ever ischemic stroke by assessing the early change in NIHSS after 2 days and 1 week and the change in mRS after 1 week and 3 months, we started the antiplatelet treatment within 9 h of onset, as the ischemic penumbra may remain viable for up to 12 h [6].

Along with the current clinical trial, the efficacy and safety of 180-mg loading dose of ticagrelor administered within 9 h of first-ever ischemic stroke compared to aspirin were assessed through NIHSS, mRS, duration of hospital stay, and possible hemorrhagic complications.

## Methods

### Sample size

This pilot study was based on the study carried out by Johnston and colleagues (2016) [7]. We used Epi Info STATCALC to calculate the sample size by considering the following assumptions: 95% two-sided confidence level, with a power of 80%, alpha error of 5%, and odds ratio calculated = 1.115. The final maximum sample size, which was taken from the Epi Info output, was 156. We increased the sample size to 169 patients to assume any dropout cases during the follow-up period. *A* total of 169 patients continued to follow up along the entire study timeline, of which 85 patients received ticagrelor and 84 patients received aspirin.

We conducted our open-label randomized prospective controlled trial between May 2019 and September 2020 after approval of the ethical committee of the faculty of medicine at Ain Shams University and the ethical committee of the faculty of medicine Kafr El-Sheik University.

We got formal written informed consent from all eligible patients or their first order of kin before randomization.

We used a web-based centralized blocked randomization plan to randomly allocate patients in a one-to-one ratio to receive ticagrelor or aspirin. Still, all of the clinical investigators were blind to the block size of the randomization plan, but the patients were aware of the antiplatelet used in the study.

The study was composed of two parallel groups: the ticagrelor group, which received a 180 mg loading dose during the first 9 h of stroke onset followed by 90 mg b.i.d from the second to the 90th day, and the aspirin group, which received a 300 mg loading dose during the first 9 h of stroke onset followed by 300 mg q.d. from the 2nd day to the 14th day then 75 mg q.d. from the 15th day to the 90th day.

This analysis was specifically designed as a pilot study to examine the preliminary efficacy, safety, and feasibility of pursuing a large-scale randomized clinical trial powered properly for safety and efficacy.

### Inclusion criteria

We included both genders with eligible ages ranging between 18 and 75 years, with the first-ever presentation with acute non-cardioembolic ischemic stroke diagnosed by appropriate clinical history, examination, and specific brain imaging findings. Patients with previous transient ischemic attacks (TIA) were not excluded from the study. Patients are not eligible for rt-PA treatment, as rt-PA might lead to hemorrhagic complications and bias our assessment of the safety of the trial medications. Also, In patients who are treated with rt-PA, initiation of antiplatelet agents should be delayed until after 24 h post-thrombolysis [8].

### Exclusion criteria

We excluded patients who had not been followed up on for 90 days after enrollment, those with NIHSS less than three or more than 25 or who had rapidly resolving symptoms before imaging results, patients with a known history of persistent or recurrent CNS pathology (e.g., epilepsy, meningioma, multiple sclerosis, history of head trauma with a residual neurological deficit).

We excluded patients who had a cardioembolic ischemic stroke before starting treatment or retrogradely. We considered ischemic stroke a cardio-embolic one when the patient had major or minor risk factors of having a cardiac source of embolus as mechanical cardiac valves, atrial fibrillation, mitral valve prolapse, aortic valve stenosis or calcification, and patent foramen ovale [9, 10], and we considered the patient to have clinical AF when his standard 12-lead ECG recording had at least 30 s showing heart rhythm with no discernible repeating P waves and irregular RR intervals (when atrioventricular conduction is not impaired) [11].

We ruled out patients who had already recurrent stroke before enrollment in our trial diagnosed by appropriate clinical history and/or MRI brain findings, as those patients might receive antiplatelet treatment as secondary prevention and might have a residual neurological deficit which could bias the assessment of safety and efficacy of the antiplatelet in our trial.

We excluded patients who had clinical seizures at the onset of their stroke, as well as those who had symptoms of any major organ failure, active malignancies, or an acute myocardial infarction within the previous 6 weeks, and those who were on warfarin or new oral anticoagulants, regular ticagrelor during the week before admission, or chemotherapy within the previous year.

For safety measures and to avoid associated confounders, we excluded patients with active peptic ulcers, GIT surgery, bleeding history within the last year, and those with a history of major surgery within the last 3 months.

We ruled out of our trial patients who had a known allergy to the study drugs and those with INR > 1.4, P.T. > 18, blood glucose level < 50, > 400 mg/DL, blood pressure < 90/60, > 185/110 mmHg on admission, or platelets < 100,000.

We considered pregnant and lactating patients or those with stroke due to venous thrombosis and stroke following cardiac arrest, or profuse hypotension ineligible for our trial.

### Study procedures

We collected the following data: age, sex, medical history of hypertension (HTN), diabetes mellitus (DM), ischemic heart disease (IHD), hyperlipidemia, tobacco use, and the time from symptoms onset to the start of treatment.

We diagnosed ischemic stroke based on the specific clinical history and examination and MRI brain using stroke protocol: T1W, T2W, FLAIR, DWI, T2 echo gradient, and MRA of all intra-cerebral vessels.

Before randomization, all the patients assessed for eligibility to participate in the trial underwent 12-lead routine ECG and transthoracic echocardiography, and immediately after randomization and receiving loading antiplatelet treatment, every patient enrolled in our trial underwent 24 h of continuous cardiac rhythm monitoring and transesophageal echocardiography, and we excluded patients with major or minor risk factors for cardio-embolic stroke.

All the patients in our study underwent carotid duplex and baseline laboratory investigations (lipid profile, liver functions, coagulation profile, complete blood count, and blood sugar).

We estimated the safety of loading ticagrelor by looking at hemorrhagic complications assessed using the PLATO bleeding definition [12].

Hemorrhagic transformation of the infarct was determined by performing a follow-up CT brain scan after 2 days and after 1 week or discharge to detect the hemorrhage; additionally, the European Cooperative Acute Stroke Study (ECASS) classification [13] was used to detect the type of hemorrhagic transformation.

We evaluated clinical improvement using five factors: the first two factors assessed the difference between NIHSS at baseline and the 2nd day and the difference between NIHSS at baseline and (the 7th day of discharge) in each patient. A decrease of four points or more in the NIHSS score was considered a significant improvement [14].

The third and fourth factors assessed mRS after 1 week of discharge and after 90 days, and all of our patients had baseline mRS of zero. mRS ≤ 2 was considered a favorable outcome [15, 16].

The fifth factor was the total number of days each patient spent in the hospital.

### Primary endpoint

Safety of ticagrelor (180 mg loading dose during the first 9 h of stroke onset followed by 90 mg twice daily from the 2nd to the 90th day given orally) vs aspirin (300 mg loading dose during the first 9 h of stroke onset followed by 300 mg q.d. from the 2nd day to the 14th day then 75 mg q.d. from the 15th day to the 90th day) regarding hemorrhagic complications using the PLATO bleeding definition, and was assessed by rates of patient suffered from hemorrhagic complications in each group.

### Secondary endpoint

The clinical outcome of the patients (assessed by rates of a favorable outcome with [NIHSS] decrease by 4 points or more on the 2nd day of admission and the 7th day of discharge compared to baseline), and (rates of a favorable outcome with mRS 0–2 after 1 week or discharge and after 90 days in a face-to-face interview in the outpatient clinic).

### Statistical analysis of the data

All efficacy and safety analyses were based on the intention-to-treat principle, and we analyzed our data using the IBM SPSS software package, version 20.0 (Armonk, NY: IBM Corp.). Statistical analysis was performed for the primary and secondary endpoints separately.

We described numerical data as means ± S.D. or median and interquartile range **[**IQR]), depending on their distribution, assessed by the Shapiro–Wilk test, and we expressed categorical data using numbers and percent.

We used Pearson’s chi-square to correlate categorical data and the Mann–Whitney *U* test to compare the abnormally distributed numerical data.

## Results

**Fig. 1:**
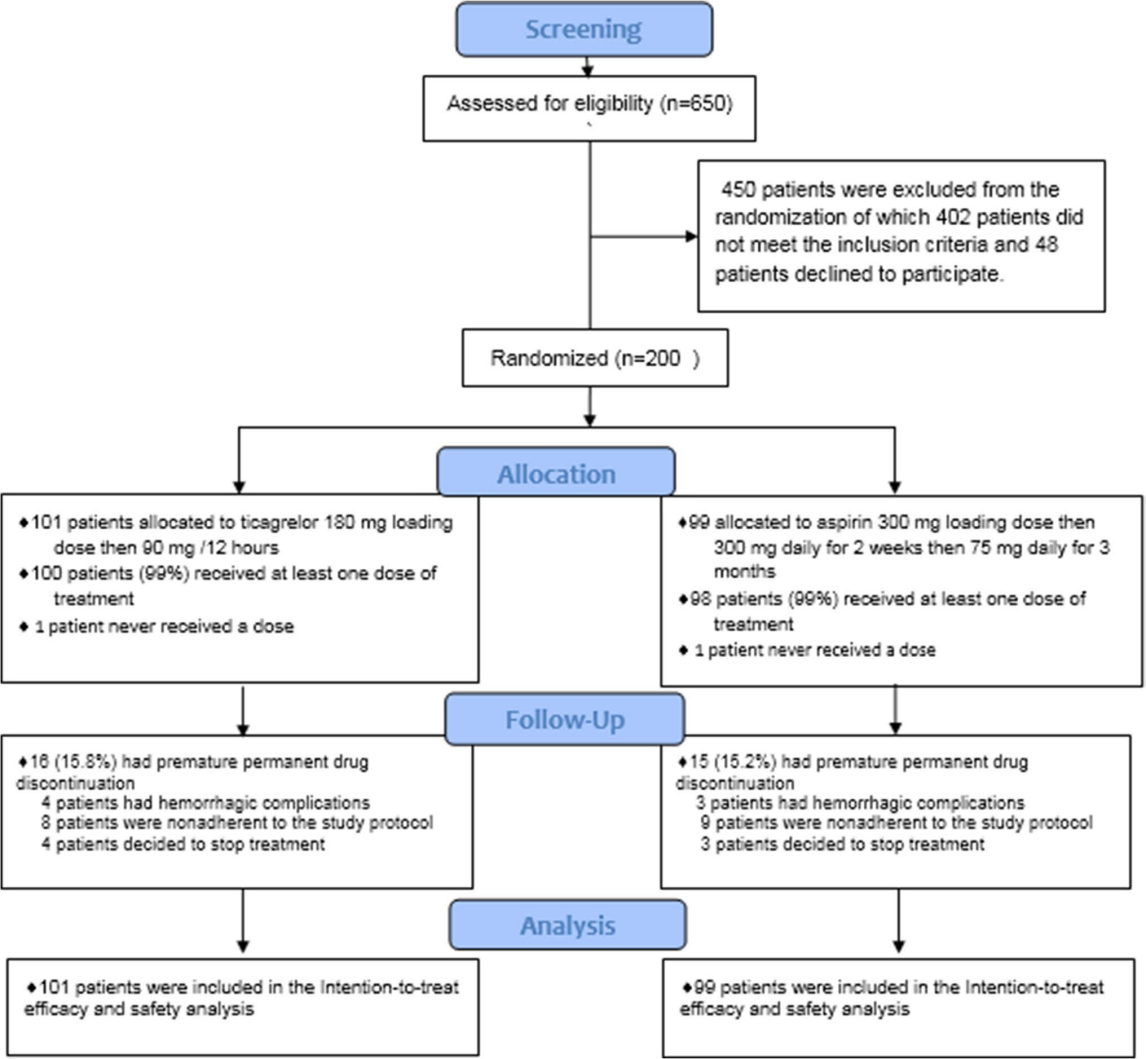
Study flow diagram

Overall, 650 patients were screened for eligibility; 200 patients (132 males and 68 females) underwent randomization and were divided into two parallel groups. The aspirin group consisted of 99 patients, and the ticagrelor group consisted of 101 patients; a total of 169 patients (113 males and 56 females) completed the pilot study during the 3-month follow-up period, as shown in Fig. 1.

There were no statistically significant differences between the two study arms regarding the baseline characters, as shown in Table 1.

**Table 1:** Baseline criteria of participants

Character	Ticagrelor arm (*n* = 101)	Aspirin arm (*n* = 99)	*P*-value
Age, median (IQR)†	**62 (60–67)**	**60.8 (59–64)**	**0.29**
Male, no. (percent) *	**68 (67.3%)**	**64 (64.6%)**	**0.77**
Time till receiving treatment, median (IQR) †	**7 (6–9)**	**7 (7–8)**	**0.58**
Medical history, no. (percent) *			
Smoker	**59 (58.4%)**	**62 (62.6%)**	**0.57**
Dyslipidemia	**49 (48.5%)**	**46 (46.5%)**	**0.78**
Diabetes mellites	**33 (32.7%)**	**31 (31.3%)**	**0.88**
Hypertension	**52 (51.5%)**	**49 (49.5%)**	**0.89**
IHD	**73 (72.3%)**	**68 (68.7%)**	**0.64**
Baseline NIHSS, median (IQR) †	**10 (9–14)**	**10 (9–12)**	**0.51**
Stroke vascular territory according to brain imaging*			
MCA	**53 (52.5%)**	**51 (51.5%)**	**1.0**
ACA	**8 (7.9%)**	**6 (6.1%)**	**0.79**
PCA	**8 (7.9%)**	**6 (6.1%)**	**0.79**
Cerebellar	**6 (5.9%)**	**8 (8.1%)**	**0.60**
Brain stem	**11 (10.9%)**	**10 (10.1%)**	**1.0**
One territory	**15 (14.9%)**	**18 (18.2%)**	**0.58**
TOAST classification *			
Small vessel	**43 (42.6%)**	**40 (40.4%)**	**0.78**
Undetermined	**20 (19.8%)**	**23 (23.2%)**	**0.61**
Large vessel	**38 (37.6%)**	**36 (36.4%)**	**0.88**

^*†*^, median; *IQR*, interquartile range; *, percentage; *IHD*, ischemic heart disease; *MCA*, middle cerebral artery; *ACA*, anterior cerebral artery; *PCA*, posterior cerebral artery; *TOAST*, Trial of ORG 10,172 in acute stroke treatment

Hemorrhagic transformation of infarction inflicted two patients (2%) in the aspirin group; one had ECASS HI type 1. The other had ECASS HI type 2. In comparison, in the ticagrelor group, one patient (1%) had hemorrhagic infarction (ECASS HI type 1). Minor bleeding (hematuria) occurred in two patients (2%) in the aspirin group and three patients (3%) in the ticagrelor group, with no statistically significant differences between the two groups, as shown in Table 2.

**Table 2:** Association between antiplatelet type and hemorrhagic complications

Hemorrhagic complications	Aspirin group (*n* = 99)		Ticagrelor group (*n* = 101)		*P*-value
	No.	%	No.	%	
Hemorrhagic transformation of infarction *	**2**	**2.0%**	**1**	**1.0%**	**0.62**
Major and minor bleeding *	**3**	**3.0%**	**4**	**4.0%**	**1.0**

^*^, percentage

Regarding clinical outcomes, there were no statistically significant differences between the two study arms regarding the decrease in NIHSS score after 2 days, but in the ticagrelor arm, 58 (57.4%) patients showed significant improvement regarding the decrease in NIHSS score after 1 week or discharge compared with 35 (35.4%) in the aspirin group with a *P*-value (< 0.01), as shown in Table 3.

**Table 3:** Association between the antiplatelet type and clinical outcomes

Clinical outcomes	Ticagrelor arm (*n* = 101)	Aspirin arm (*n* = 99)	*P*-value
Significant improvement in NIHSS after 2 days*	**40 (39.6%)**	**27 (27.3%)**	**0.07**
Significant improvement in NIHSS after 1 week or discharge*	**58 (57.4%)**	**35 (35.4%)**	< 0.01**
Favorable outcome mRS (0–2) after 1 week or discharge*	**27 (26.7%)**	**14 (14.1%)**	0.04**
Favorable outcome mRS (0–2) after 90 days*	**39 (38.6%)**	**23 (23.2%)**	0.02**
Days of hospital stay, median (IQR) †	**8 (7–9)**	**8 (7–11)**	**0.24**

^*†*^, median; *IQR*, interquartile range; *, percentage; *NIHSS*, National Institute of Health Stroke Scale; *mRS*, modified ranking scale; **, statistically significant at *P*-value < 0.05. Significant improvement in NIHSS implies a reduction in NIHSS by 4 points or more

Regarding mRS, 27 (26.7%) and 39 (38.6%) patients in the ticagrelor group showed favorable mRS scores after 1 week or discharge and after 90 days, respectively, compared with 14 (14.1%), 23 (23.2%) in the aspirin group with *P*-value (0.04 and 0.02), as shown in Table 3.

## Discussion

There is an increasing need for new antiplatelets with better efficacy in managing ischemic stroke patients, especially those who do not fit rt-PA therapy secondary to its relatively short window (4.5 h of symptom onset) and its relatively high price and low accessibility, especially in developing countries. [2].

Regarding the current clinical trial’s primary endpoint, there was no statistically significant difference between the two groups regarding hemorrhagic complications. This result agrees with the results of Johnston and colleagues (2016), Wang and colleagues (2017), and Amarenco and colleagues (2017), who found that there was no statistically significant difference between ticagrelor and aspirin as regards hemorrhagic infarction [7, 17, 18].

Concerning our secondary endpoint, we found that patients who received ticagrelor showed statistically significant clinical improvement regarding NIHSS score after 2 days and 1 week of discharge and more favorable mRS scores after 1 week of discharge and after 90 days compared with patients who received aspirin.

Even though there has been no such study that compared the outcome of ischemic stroke in patients receiving, loading ticagrelor, or loading aspirin within the first 9 h of onset, our results can be partially in agreement with Wang and colleagues (2017), Amarenco and colleagues (2017), and Johnston and colleagues (2020), who found that patients who received ticagrelor had better clinical outcomes than those receiving aspirin results [5, 17, 18].

Our results may be that ticagrelor is a potent P2Y12 adenosine diphosphate platelet receptor inhibitor that decreases platelet activation in the acute phase of ischemic stroke, which prevents the release of neurotoxic and thrombogenic eicosanoids, including thromboxane B2, and, as a result, it might reduce early death and improve outcomes in survivors by reducing the volume of brain penumbra and reducing the risk of early recurrent ischemic stroke and pulmonary embolism Van Kotte and colleagues (1994) and Wallentin and colleagues (2009) [19, 20].

Although our results were encouraging, our pilot study has some limitations: first, the sample was intended to establish the feasibility of a larger-scale trial powered for both safety and efficacy, and this nature of the sample limits the validity and generalizability of the result; second, patients were not blind to the treatment they received.

## Conclusion

Compared to aspirin, ticagrelor had a better clinical outcome based on NIHSS and mRS on acute ischemic stroke patients who received it within 9 h from symptom onset and had a less hospital stay duration. Ticagrelor was non-inferior to aspirin regarding hemorrhagic complications.

## Data Availability

The datasets generated and analyzed during the current study are not publicly available due to the ethical regulations of our university but are available from the corresponding author (Mohamed G. Zeinhom) on reasonable request.

## Abbreviations

*ADP*: Adenosine diphosphate
CNS: Central nervous system
*CT*: Computerized tomography
*ECASS*: European cooperative acute stroke study
*ECG*: Electrocardiogram
*FDA*: Food and Drug Administration
*GIT*: Gastrointestinal tract
*INR*: International normalization ratio
*IQR*: Interquartile range
*MRI*: Magnetic resonance imaging
*mRS*: Modified Rankin scale
*NIHSS*: National Institute of Health Stroke Score
*rTPA*: Recombinant tissue plasminogen activator
*TIA*: Transient ischemic attack
